# A Diagnostic and Performance System for Soccer: Technical Design and Development

**DOI:** 10.3390/sports13010010

**Published:** 2025-01-08

**Authors:** Alberto Gascón, Álvaro Marco, David Buldain, Javier Alfaro-Santafé, Jose Victor Alfaro-Santafé, Antonio Gómez-Bernal, Roberto Casas

**Affiliations:** 1 Aragon Institute of Engineering Research, University of Zaragoza, 50018 Zaragoza, Spain; algaroche@unizar.es (A.G.); buldain@unizar.es (D.B.); rcasas@unizar.es (R.C.); 2 Centro Universitario de la Defensa, Academia General Militar, 50090 Zaragoza, Spain; 3 Podoactiva Research Department, 22197 Cuarte, Spain; javieralfaro@podoactiva.com (J.A.-S.); victoralfaro@podoactiva.com (J.V.A.-S.); antoniogomez@podoactiva.com (A.G.-B.); 4 Podiatrist of the Spanish National Football Team, 28036 Madrid, Spain; 5 Podiatrist of Real Madrid CF, 28036 Madrid, Spain; 6 Podiatrist of SD Huesca, 22004 Huesca, Spain

**Keywords:** wearable technology, machine learning algorithms, inertial sensors, soccer biomechanics, foot kinematics

## Abstract

This study presents a novel system for diagnosing and evaluating soccer performance using wearable inertial sensors integrated into players’ insoles. Designed to meet the needs of professional podiatrists and sports practitioners, the system focuses on three key soccer-related movements: passing, shooting, and changes of direction (CoDs). The system leverages low-power IMU sensors, Bluetooth Low Energy (BLE) communication, and a cloud-based architecture to enable real-time data analysis and performance feedback. Data were collected from nine professional players from the SD Huesca women’s team during controlled tests, and bespoke algorithms were developed to process kinematic data for precise event detection. Results indicate high accuracy rates for detecting ball-striking events and CoDs, with improvements in algorithm performance achieved through adaptive thresholds and ensemble neural network models. Compared to existing systems, this approach significantly reduces costs and enhances practicality by minimizing the number of sensors required while ensuring real-time evaluation capabilities. However, the study is limited by a small sample size, which restricts generalizability. Future research will aim to expand the dataset, include diverse sports, and integrate additional sensors for broader applications. This system offers a valuable tool for injury prevention, player rehabilitation, and performance optimization in professional soccer, bridging technical advancements with practical applications in sports science.

## 1. Introduction

The use of sensors for monitoring in the world of sport, and especially in soccer, generates great interest due to its potential for improving sports performance, analyzing players’ movements [1,2], and lowering the injury risk through the assessment of physical loads borne by each player [3,4]. Moreover, these technologies play an important role in rehabilitation processes, providing objective metrics to check the health status of the player [5,6].

Despite these advancements, there is a critical need to bridge the gap between sports performance analysis and clinical applications. Many musculoskeletal injuries in athletes arise from repetitive biomechanical stresses, which can be identified and mitigated through precise kinematic analysis [7]. A clinical approach, therefore, complements traditional performance monitoring by offering tools to optimize biomechanics and reduce injury risks. This dual focus allows the system to serve not only coaches and sports scientists but also clinicians aiming to improve the health and functionality of athletes.

Currently, the challenges of applying sensors to collective sport scenarios, including collisions and unpredictable movements, have led to most of the work in this field being focused on individual player monitoring [8]. Players commonly wear vests equipped with sensors that monitor their speed, position, distance traveled and heart rate. While video-based systems are also widely used for monitoring actions, their implementation involves high costs due to the necessity of multiple cameras to ensure full coverage and avoid line-of-sight occlusions [9]. In addition, the identification of events such as passes or shots requires the use of artificial intelligence (AI) or manual labeling by personnel, both of which are resource-intensive in terms of cost and time [10,11].

Event monitoring through the identification of specific gestures like passes, shots, or dribbles provides valuable insights for both individual and team performance improvement. Systems like ProZone [2] and Opta [12] are widely recognized professional tools that deliver high-quality information, leveraging camera-based systems to track players and events. However, the aforementioned constrains limit their accessibility and practicality, especially for semi-professional and amateur teams.

In contrast, inertial sensor-based systems for event detection, like the one presented in this study, provide a cost-effective and adaptable solution. These systems eliminate line-of-sight issues by embedding sensors directly into players’ equipment, ensuring uninterrupted data collection even in complex scenarios [10]. Inertial sensors can identify events such as passes and shots in real-time using embedded algorithms or cloud architectures, reducing the need for manual intervention or extensive AI training. Furthermore, their portability and non-intrusive design allow for use in matches, training sessions, or doctor consultations, offering immediate feedback. This real-time capability, combined with lower costs and simplified implementation, positions inertial sensors as a practical alternative for teams at various competitive levels.

Despite the potential advantages of this type of approach, research employing inertial sensors for soccer-specific remains limited. Most of them focus on the detection of passes and shots, as these are the primary events of interest. For instance, ref. [4] utilized five IMUs placed on the shins, thighs, and sacrum to estimate physical loads during training and matches. Similarly, ref. [13] employed neural networks to identify passing, shooting, and running movements using a comparable five-sensor setup. On the other hand, ref. [10,14] demonstrated the feasibility of using a single sensor located in the insole to detect passes, shots, and instep strikes in controlled scenarios. More recently, ref. [11] extended this approach to real-match conditions, focusing on pass and shot detection. Additional innovations include the use of heel-mounted sensors for strike detection [15] and textile pressure matrix sensors embedded in soccer shoes to evaluate various types of kicks [16]. Studies like [17,18] have also utilized inertial sensors (one to five units) to estimate physical loads during matches.

From the review of existing IMU-based systems, it is evident that significant progress has been made, particularly in reducing the number of required sensors and implementing neural network models capable of recognizing diverse events. However, several limitations persist. Firstly, comfort remains a crucial concern for wearable design [19], but many systems depend on multiple body-mounted sensors, heavy external batteries, or storage units, which may interfere with the player activity. The worst-case scenario involves wired setups, further complicating their usability in dynamic sports environments. Secondly, real-time data processing remains a significant challenge, with most studies relying on post hoc analysis [10,11,13,14,15,16,17,18]. This delay in delivering actionable insights limits the practical application of such systems during training sessions, doctor consultations, or live matches, where immediate feedback is essential.

This paper addresses the limitations of current performance evaluation systems by integrating a clinical perspective into sports analysis. Biomechanical insights derived from foot kinematics are crucial not only for optimizing athletic performance but also for preventing injuries. A clinical approach enables the identification of subtle biomechanical imbalances or risk factors, which can be addressed through targeted interventions [20], aligning the system with broader applications in rehabilitation and movement optimization [21].

To achieve this, this paper presents a novel system for soccer performance evaluation that leverages inertial wireless sensors embedded in players’ insoles [22]. This approach allows for seamless usage during matches without interfering with players’ movements. Real-time evaluation is made possible through wireless communication and decentralized algorithms executed on a cloud infrastructure. By analyzing players’ foot dynamics in situ, this system provides actionable insights that benefit both performance enhancement and injury prevention, bridging the gap between sports and healthcare applications.

## 2. Materials and Methods

### 2.1. System Overview

**Figure 1:**
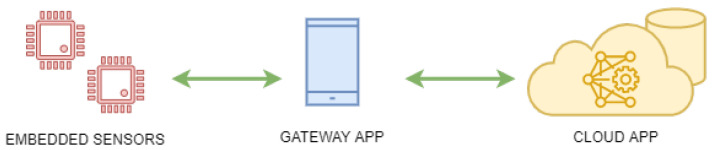
System architecture.

A comprehensive solution was developed, comprising an inertial sensor for capturing foot movement data, a cloud application for analyzing this data, and a mobile application that bridges these components while serving as the user interface (see Figure 1).

#### 2.1.1. Embedded Sensors

The core of the system is a compact sensor module designed to capture and transmit motion data without obstructing the athlete’s performance. Key features include:

- Inertial Measurement Unit (IMU): The LSM6DSOX [23], a six-axis sensor integrating a 3D accelerometer and gyroscope, captures precise motion data. Its low power consumption (550 µA) and compact dimensions (2.5 × 3.0 × 0.83 mm) make it suitable for wearable applications and outperforms alternatives like the BMI160 (Bosch) [24] and MPU-6050 (InvenSense) [25].
- Microcontroller: The EFR32BG22 from Silicon Labs [26] handles data processing and wireless communication via Bluetooth Low Energy (BLE 5.2). With a 32-bit ARM Cortex-M33 processor and low energy usage (2.6/3.6 mA in Rx/Tx), it ensures efficient data management and transmission.
- Flash Memory: A 64 Mbit MX25R6435F [27] stores motion data, supporting both real-time analysis and offline logging.
- Battery: The system is powered by a 3.7 V, 60 mAh lithium-ion battery, providing up to 20 h of continuous operation. Its rechargeable design ensures convenience and reliability for prolonged use.

**Figure 2:**
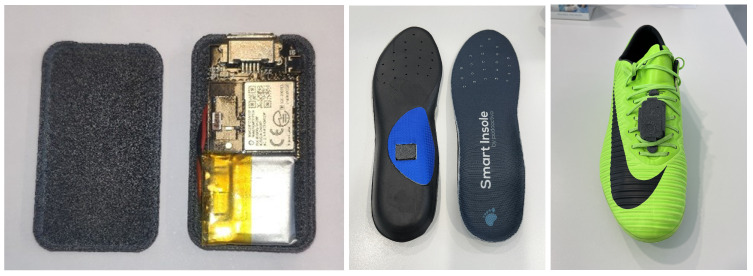
(**left**) IMU Sensor. (**middle**) IMU Sensor placed inside the insole. (**right**) IMU Sensor placed on the instep.

The entire module is housed within a 20 × 40 × 5 mm enclosure, small enough to fit inside a standard insole or on the instep, as shown in Figure 2.

#### 2.1.2. Cloud Application

**Figure 3:**
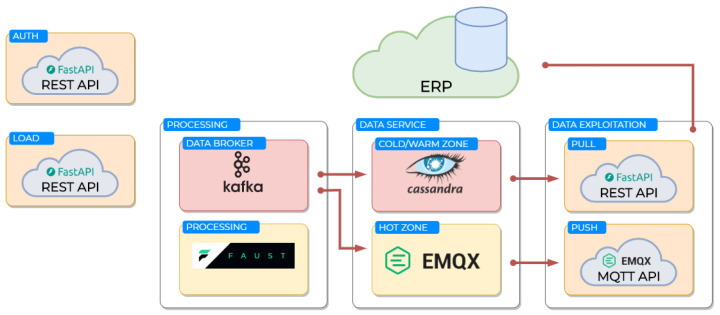
Cloud APP architecture.

A cloud platform is responsible for analyzing the data recorded by the sensor during each test and extracting relevant performance metrics for the user. The architecture of this platform is depicted in Figure 3. Data from the sensors are transmitted via the mobile app to the cloud, where they are processed using a combination of Apache Kafka [28] for messaging and Faust [29] for stream processing. This setup enables parallel data handling, ensuring efficient performance even when accommodating multiple users. The processed data are stored in an Apache Cassandra NoSQL database [30], which is optimized for efficiently managing time-series data. A REST API developed with FastAPI [31] allows seamless integration with external systems, while an MQTT broker [32] notifies the mobile app once the analysis is complete.

#### 2.1.3. Mobile App

**Figure 4:**
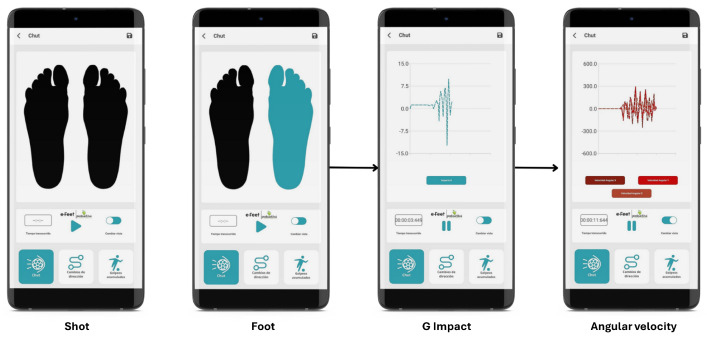
View of different screens of the mobile APP.

A mobile application, designed for seamless user interaction, serves as an interface between the sensors and the cloud (Figure 4). The application enables users to configure tests and view live motion data from the sensors, streaming this data to the cloud in real time for analysis. Once the cloud processing is complete, the app retrieves and displays the results in a user-friendly format, facilitating user-centric decision-making. Additionally, the app integrates with an external ERP system to securely manage user data and link trials to individual users.

### 2.2. Data Exchange

**Figure 5:**
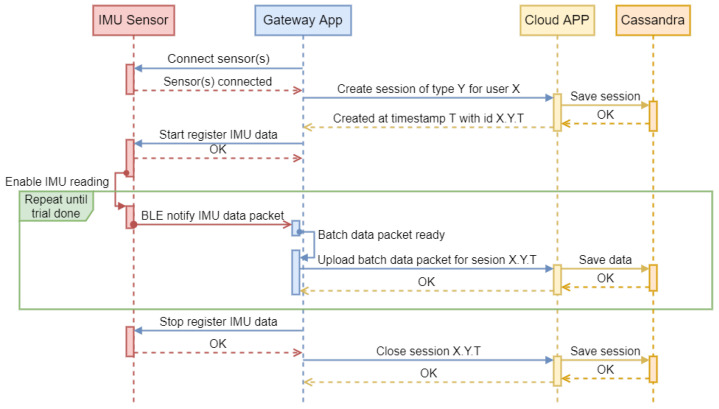
First part of the sequence diagram for session data register.

The data recording and analysis process is based on the concept of a session. When recording data for a user’s test (Figure 5), the mobile app first establishes a connection with the sensors on both feet. It then requests the cloud application to create a session for the user and test type. The cloud application creates and stores the session in the database, then sends the mobile app a session identifier, which includes the user ID, test type, and session creation timestamp. The mobile app instructs the sensors to start IMU data logging, sending acceleration and angular velocity packets every 20 ms. To prevent overloading communication with the cloud application, packets are accumulated and sent in batches along with the session identifier. The data are then saved in the database. At the end of the test, sensor data recording stops, the session is closed, and the data are ready for analysis.

**Figure 6:**
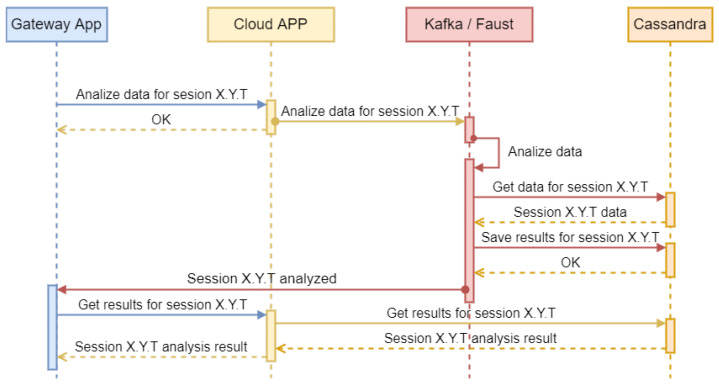
Second part of the sequence diagram for session data register.

To analyze the data (Figure 6), the mobile application requests the cloud application to analyze the session data identified by their ID. Because the analysis process can be resource-intensive depending on the test type and data volume, the process is performed asynchronously by sending a request to Kafka/Faust for background processing. Once the processing service receives the request, it retrieves the session data, performs the analysis, and saves the results to the database. The application is then notified of the analysis completion via the MQTT broker.

### 2.3. Experiment Design

**Figure 7:**
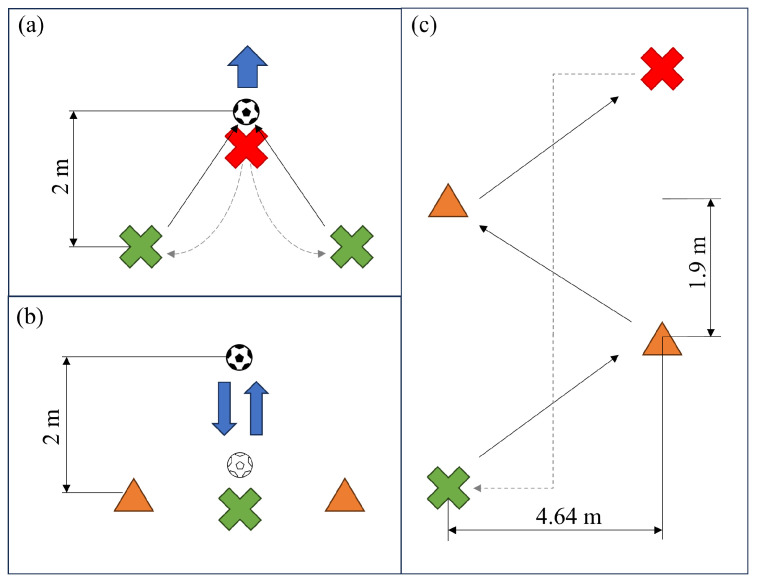
This image shows the different setups of the tests. The green mark indicates the starting point, the red mark indicates the end point and the blue arrows indicate the movement of the ball. The continuous arrows show the movement of the player during the test and the dashed arrows show the return movement to the starting point. (**a**) Shooting test setup, (**b**) passing test setup, and (**c**) change of direction test setup.

This study focuses on analyzing foot kinematics across three test types relevant to professional soccer (Figure 7): set shots, passes, and changes of direction (CoDs). The objective is to develop a clinical analysis tool for professional podiatrists. This tool helps assess player performance during training, identify potential issues, and refine their technique.

To generate a robust dataset, the tests were performed by professional players from SD Huesca, a team in the 2nd Spanish women’s division. The group included players from various positions, as well as both right- and left-footed players.

The development of this clinical tool focused on three main objectives: achieving high precision in data collection, ensuring the comfort of players during testing, and creating actionable insights that could inform clinical interventions. Each of these objectives presented specific challenges:

1. Precision in Data Collection: The kinematic data collected during the tests needed to capture even the smallest deviations in movement patterns. This required refining algorithms to accurately identify key biomechanical events such as asymmetries or abnormal loading, particularly in dynamic activities like CoD.
2. Player Comfort: Given that professional athletes were participating, it was essential to ensure the tests were minimally invasive. The integration of sensors in the insoles prioritized unobtrusiveness while maintaining accuracy. Multiple prototypes were tested to ensure that the placement of sensors did not interfere with player performance or comfort.
3. Clinical Actionability: The tool was designed to produce outputs that would provide clear, clinically relevant insights. This included developing customizable reports and dashboards that clinicians could use to assess injury risks or recommend improvements in technique.

#### 2.3.1. Shooting Test

The shooting test aims to obtain data to analyze the biomechanics of shots such as free kicks and penalty kicks. This trial considers a shot of some power after a short run, with the ball static and in a completely controlled situation in which the player can prepare with sufficient time (Figure 7a). From a clinical approach, it is essential to understand how foot placement and striking technique affect both shot efficiency and player health. The tool can identify patterns that may predispose to injury or highlight optimal techniques that minimize risk while maximizing performance.

The shooting test consists of 30 set shots with each foot. The player will start the test standing two meters away from the ball; when given the signal, she will run towards the ball and hit it, after which she will stand still for two seconds and walk back to the starting point, where she will wait a short time before repeating the movement.

Methodology for Analysis:

1. Kinematic Data Collection: The inertial sensors embedded in the insoles record angular velocities and accelerations during each shot. These parameters are processed to identify peak forces and joint angles at the moment of ball impact.
2. Event Detection Algorithms: A combination of threshold-based methods and machine learning models is used to distinguish critical events such as the plant foot stabilization and follow-through phases.
3. Clinical Interpretation: The data are analyzed to detect potential inefficiencies or patterns associated with increased injury risk, such as lateral imbalances or excessive impact forces.

#### 2.3.2. Passing Test

The passing test presents a different scenario where passing power is lower, but the movement dynamics introduce more stress due to the lack of full ball control. This requires user mobility and limits reaction and preparation times. In sports practice, it is valuable to evaluate the technique of foot contact with the ball and the biomechanical effects of different passing styles and forces. Clinically, it is important to analyze how these movements influence the accumulated impact loads on the foot and to optimize technique for injury prevention while improving passing accuracy and speed.

The passing test consists of 30 passes with each foot while the ball is in motion. A passer sends the ball to the player, who must return it without stopping and then return to the starting position (Figure 7b).

Methodology for Analysis:

1. Spatiotemporal Metrics: Metrics such as contact time, foot trajectory, and ball velocity are extracted from the sensor data.
2. Dynamic Stability Assessment: The data are analyzed to assess the player’s ability to maintain balance and control during dynamic movements, which is critical for accurate passing.

#### 2.3.3. CoD Test

The CoD test does not involve the ball and focuses on assessing agility and biomechanics during quick CoDs, which are crucial in soccer movements. Clinical analysis emphasizes foot stability, balance, and positioning on the ground, identifying potential injury risks such as sprains or muscle strains. It also examines reactivity during propulsion off the ground.

The CoD test consists of 30 CoDs to the right and left. The player starts at the beginning of a circuit and runs to a cone, where she performs a CoD to the left, then runs to another cone to perform a CoD to the right. After leaving this cone, she continues running for a few meters to the last cone. There, she stops briefly before returning to the start and waits a short time before repeating the movement (Figure 7c).

Methodology for Analysis:

1. Change of Direction Metrics: The system evaluates parameters such as reaction time, ground contact time, and foot placement angles during each CoD.
2. Stability: The insoles measure lateral stability, identifying potential imbalances.
3. Agility Scoring: A composite agility score is generated based on speed, precision, and biomechanical efficiency, which can guide training interventions.

### 2.4. Algorithm Description

#### 2.4.1. Data Preprocessing

Once the data were collected, the dataset was configured. For this purpose, the data were unified by synchronizing the recordings, as they were captured by two independent sensors. This synchronization process followed these steps:

1. Each data sample is numbered with a sequence number. Since the sampling period is constant, this allows establishing a time reference relative to the start of data capture and detecting any lost messages.
2. The absolute time reference of a signal is determined from the reception instant of the first data message from each sensor.
3. To synchronize both signals, the reception instants of the first N samples from both sensors are analyzed, and the average difference is calculated to determine the offset between the signals.
4. This offset is used to adjust the relative time base of one sensor to align it with the other, as the relative time base is used for data analysis, while the reception instants align with the sampling period.

**Figure 8:**
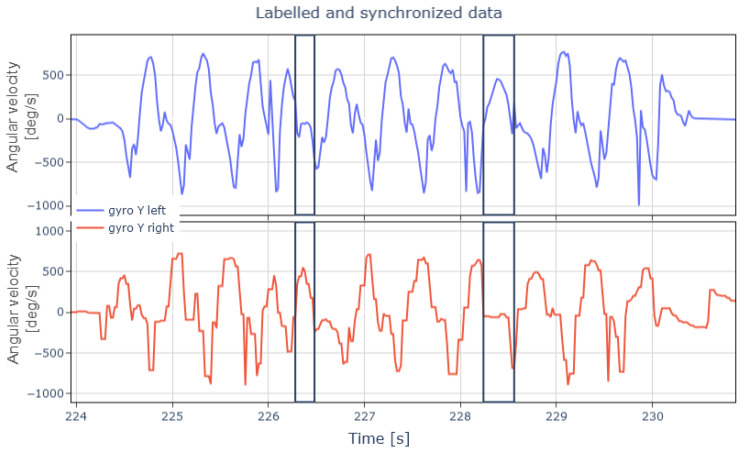
Representation of data for both feet from player 1’s labeled and synchronized change-of-direction test.

Synchronizing the data ensures access to information from both feet at any time. This synchronization is essential for all tests. In tapping tests, the supporting foot is clinically significant, but the tapping foot facilitates easier identification. In CoD tests, either foot can serve as the support foot, making both sensors critical for data collection. As shown in Figure 8, changes of direction coincide with moments when the supporting foot remains immobile longer than usual during running.

#### 2.4.2. Algorithm Description

The next step was data labeling, which involved considering the entire gesture as a stroke, from the moment the foot opposite to the striking foot is supported until the striking foot makes contact with the ground. This process was applied to both passes and shots.

**Figure 9:**
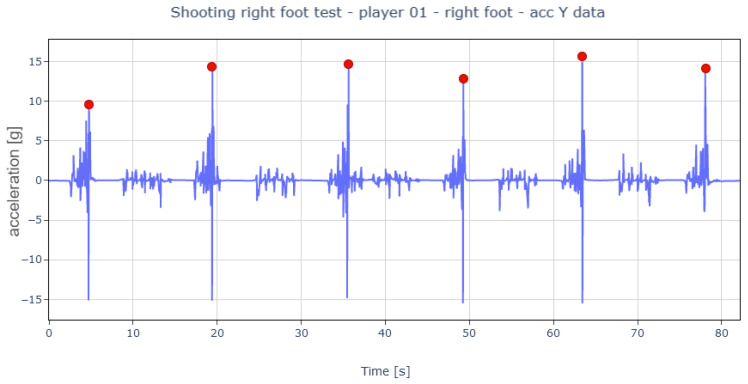
Overview of the shooting test data.

With the data labeled, algorithm development began. The *Y*-axis accelerations revealed that the moments of the stroke appeared as peaks of varying durations (Figure 9).

To analyze significant motion during the striking process, the signal was processed to quantify motion intensity (*M*) as the product of the signal’s value and its first derivative. This approach avoids relying solely on peaks, which may lead to errors due to rebounds. A moving average $\bar{M}$ was then calculated over a defined window size to smooth the data and identify trends. Using the average motion across all windows and a scaling factor (*ratio*), a threshold was established to determine whether motion was significant. A binary decision function was applied, categorizing movements as significant or not based on whether the motion exceeded the threshold.

(1) $$M_{i}=\left|x_{i}-x_{i-1}\right|\cdot x_{i}$$

(2) $${\bar{M}}_{i}=\frac{1}{size}\sum_{j=i-\frac{size}{2}}^{i+\frac{size}{2}}M_{j}$$

(3) $$Threshold=\frac{\sum_{0}^{n}{\bar{M}}_{i}}{n\cdot ratio}$$

(4) $$SignificantMovement_{i}=\left\{\begin{array}{c} 1\Rightarrow {\bar{M}}_{i}<Threshold\\ 0\Rightarrow {\bar{M}}_{i}>Threshold \end{array}\right.$$

where:
*M* = amount of motion;*i* = index of each measurement;*n* = total number of windows analyzed;size = number of elements in each window for the moving average;ratio = dimensionless factor to scale the threshold.

**Figure 10:**
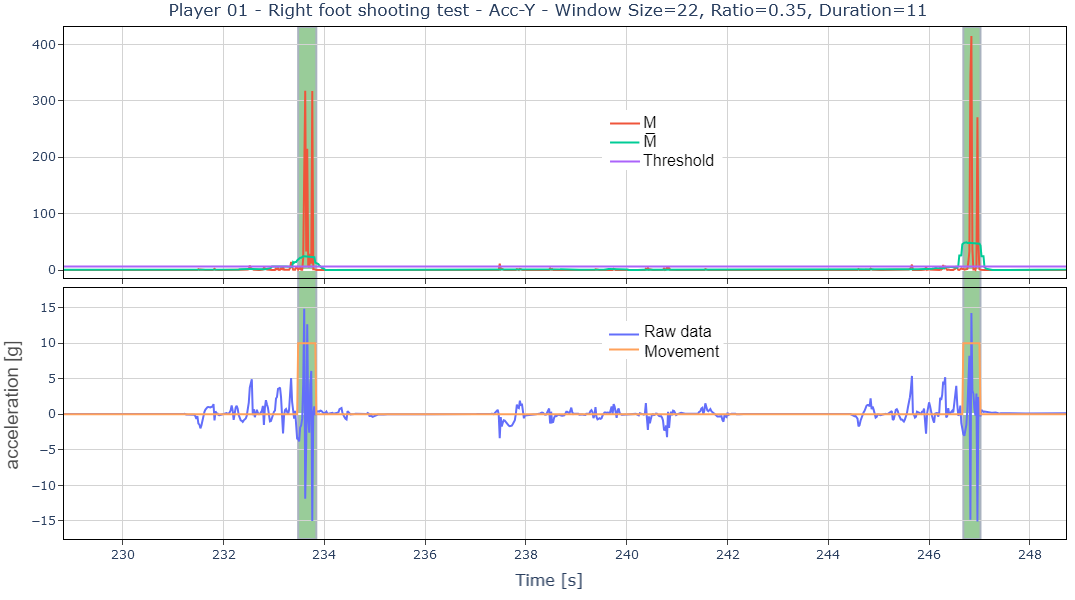
Example of the process followed by the algorithm to detect shooting events.

**Figure 11:**
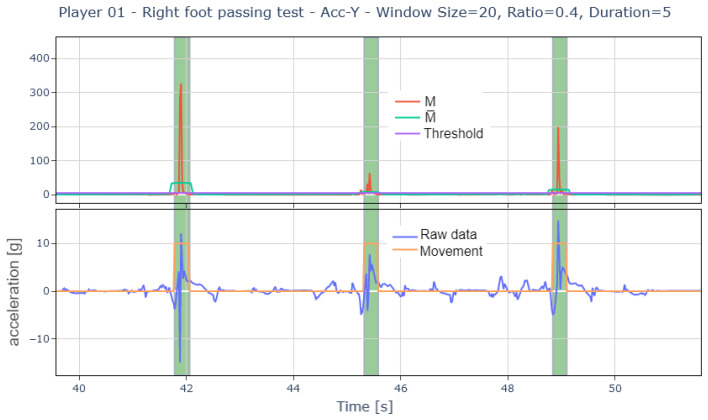
Example of the process followed by the algorithm to detect passing events.

**Figure 12:**
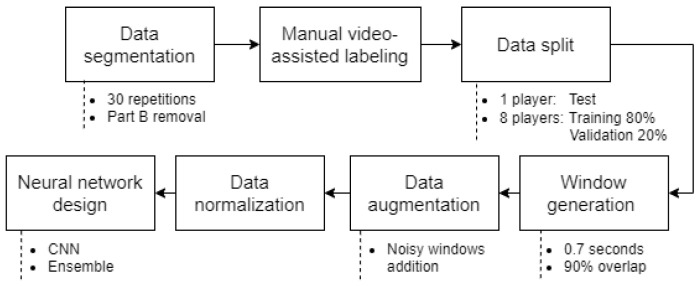
Diagram of the data preparation process.

Once significant movement areas are identified, a filtering process is applied, eliminating all events with a duration shorter than a predefined parameter. Thus, the detection of shots and passes is as follows (Figure 10 and Figure 11):

For the hit and pass tests, significant motion detection algorithms were sufficient to identify events. However, for detecting CoDs, more advanced neural network-based algorithms were required. This process is significantly more complex than the previous ones and involves extensive data processing. Before utilizing the neural networks, it was essential to prepare the data (Figure 12) to ensure their suitability for the subsequent training process. Each of these phases will be explained below.

**Figure 13:**
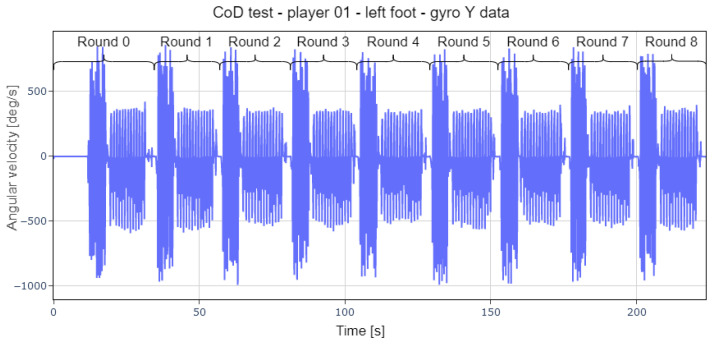
Raw data representation of a change-of-direction test file with eight rounds indicated.

The first step was the segmentation of the data, since, in order to speed up the data collection, each player performed the 30 repetitions of the test followed by short pauses in between. For this purpose, each player was assigned an identifier and her 30 rounds of each exercise were labeled (Figure 13).

**Figure 14:**
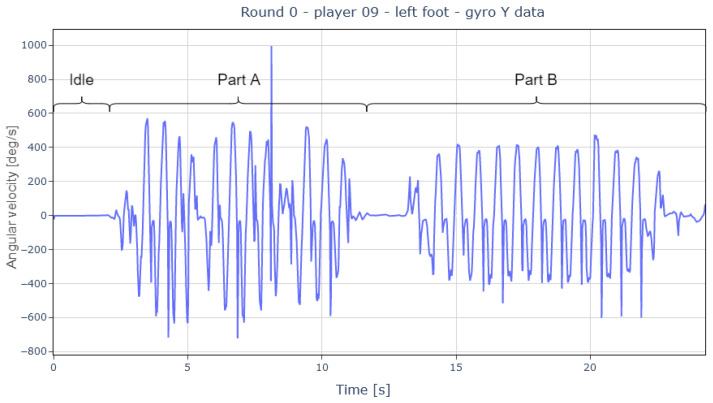
Representation of the first round of the CoD test and its different parts.

A closer look at the first round of the CoD test (Figure 14) reveals two distinct zones with movement. These zones correspond to moments when the players were running (part A) and moments when they were walking back to the starting point (part B). The segmentation process was performed using the significant motion detection algorithm described previously, as the pauses between part A and part B made it possible to differentiate them. This also allowed for the exclusion of the B parts of each repetition and the stationary parts, as they did not provide data of interest.

The next step consisted of manually labeling the data, since the exact point at which the CoD occurred was not available. For this purpose, the videos taken from all the trials were used and COD was considered as the movement between the player placing the support foot on the ground near the cone until she lifted it to continue the run.

Once labeled, three subsets were generated for training, validation, and testing. The test subset includes all data from a single player, while the training and validation subsets include data from the remaining eight players. In this way, the system would not have any information from her before processing. Of the remaining data, 20% was used for validation and 80% for training, randomly distributing the different rounds of each player.

The algorithm aims to identify time series samples corresponding to CoD events. To achieve this, the sensor data are segmented into windows, which act as snapshots of consecutive data samples, and these are then fed into the neural model. Based on an analysis of the sizes of CoD events and testing various window lengths and overlap percentages, windows of 0.7 s (at a sampling frequency of 50 Hz) with a 90% overlap were selected. As for the variables used, the Y and Z components of the gyroscope and the Z component of the accelerometer from both feet were chosen, as these exhibited the most significant variation during changes of direction.

When generating them, they needed to be classified as windows with a CoD event or without one. The criterion used to determine whether a window contained an event was that it must include at least 30% of the complete event data samples.

It was found that there were a very small number of CoD event windows compared to non-event windows, and to address the imbalance between CoD event windows (approximately 10% of total events) and non-event windows, data augmentation techniques were applied. This involved adding random sensor noise, modeled as a normal distribution with zero mean and standard deviation σ, extracted from idle moments when players were stationary. This process increased the ratio of CoD events to total events to approximately 50%, ensuring a better representation in the dataset. Additionally, data from all sensors were normalized to prevent those with larger scales (e.g., gyroscope) from dominating the neural network’s training. Care was taken to normalize the training, validation, and test subsets separately to avoid data leakage that could artificially inflate model performance.

For neural network design, convolutional neural networks (CNNs) were chosen because of their ability to capture temporal patterns in time series data, outperforming standalone fully connected architectures. The final architecture (Table 1) consisted of two convolutional layers (32 and 64 filters, kernel size 3, stride 1, ReLU activation, no padding) followed by a dense layer with 24 neurons, an intermediate dropout layer to reduce overfitting, and a final dense layer with 12 neurons to capture non-linear patterns.

To maximize performance with limited data, an ensemble model comprising eight networks was implemented, with each network trained on data excluding a different test player. This ensemble approach allowed less powerful individual models to combine their outputs, creating a more robust final prediction system. Finally, due to the typical duration of a change of direction, detections lasting less than 0.1 s were filtered out.

## 3. Results

**Figure 15:**
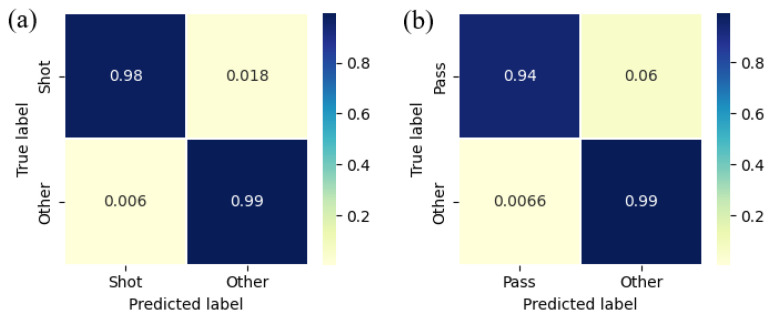
(**a**) Confusion matrix of the shooting test. (**b**) Confusion matrix of the passing test.

In the case of the kicking event detection, the main difficulty of this detection process lies in carrying out a first processing that emphasizes the areas of interest and in finding the appropriate parameters (window size, ratio, and event duration) to be able to identify the events taking into account the inherent variability of each player. In this case, a compromise between accuracy and generalization was reached to achieve a reasonably good accuracy for all the cases studied, as shown by the confusion matrices of both tests (Figure 15).

**Figure 16:**
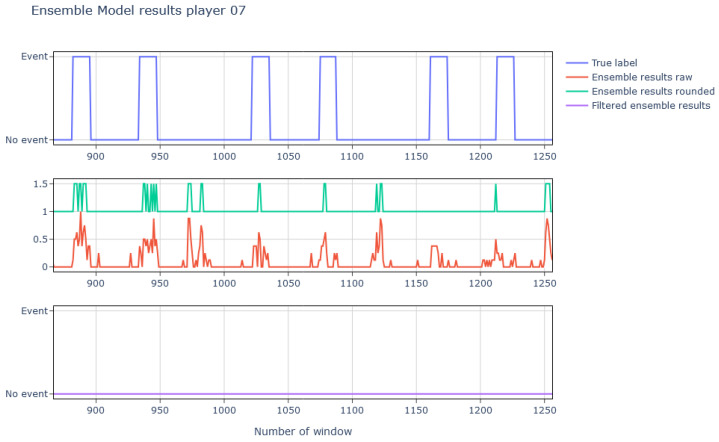
Representation of the true labels, the results of the ensemble model (the raw averaged results in red and the rounded averaged results in green), and the final predictions after filtering out those with insufficient duration.

Regarding the CoDs, nine different ensemble models were generated, trained, and tested with their corresponding test player. After applying the aforementioned filtering, the results were as shown in Figure 18. As it can be seen, the accuracies at the output of the ensemble model are quite good and even improve in some cases after applying the final filtering. However, the results obtained in complicated players such as players 6 or 7 are significantly worse and even worsen when filtering; this is because the models have more difficulties to identify the CoDs and this results in consecutive isolated detections Figure 16.

A CoD is composed of several consecutive event windows, but the model does not take previous results into account for its prediction. The final prediction (AP) is calculated as the average of the predictions from the eight models ($X_{ij}$), where 0 represents a non-event and 1 represents an event. To incorporate prior results, these AP values are smoothed using a moving average, generating the Windowed Averaged Prediction (WAP), calculated over a defined window size (*size*). The WAP reduces noise and provides a stable measure for comparison against a dynamic threshold, instead of relying on the peaks observed in Figure 16. Finally, the change of direction (CoD) event is identified when the WAP exceeds this *Threshold*, with detections lasting less than 0.1 s filtered out to remove spurious peaks. Variables such as *Threshold* and WAP inherit the same unit as the predictions ($X_{ij}$), which represent normalized probabilities between 0 and 1.

(5) $$AP_{j}=\frac{1}{7}\sum_{i=0}^{7}X_{ij},$$

(6) $$WAP_{k}=\frac{1}{size}\sum_{j=k-\frac{size}{2}}^{k+\frac{size}{2}}AP_{j},$$

(7) $$Threshold=\frac{1.75}{n}\sum WAP_{k},$$

(8) $$CoD_{k}\left\{\begin{array}{c} 1\Rightarrow WAP_{k}>Threshold\\ 0\Rightarrow WAP_{k}<Threshold \end{array}\right.$$

where:
*i* = number of model;*j* = number of predictions;*k* = number of window;*n* = total number of windows;size = number of elements in a window.

**Figure 17:**
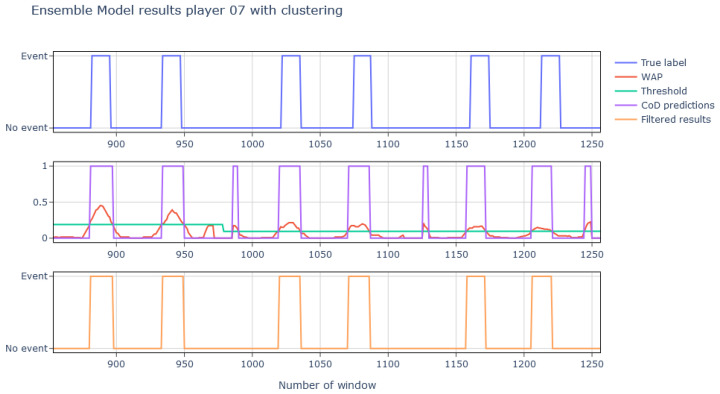
Representation of the true labels, the processing made after the ensemble model results (the threshold in green varies since test rounds have been processed separately), and the results obtained after the final filtering process.

The effect of this post-processing clustering applied to the ensemble results is shown in Figure 17, where it can be seen how the isolated events get removed.

**Figure 18:**
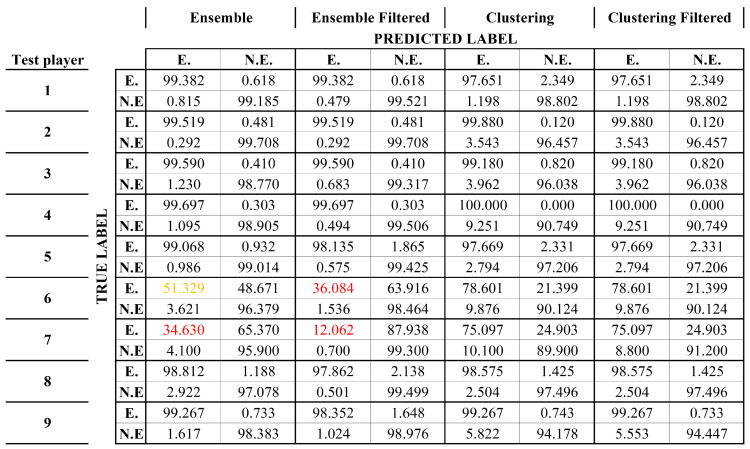
Confusion matrices of the 9 different models generated (E., event; N.E., no event). Diagonal values below 0.5 are shown in red and values below 0.7 are shown in orange.

Looking at Figure 18, it can be seen how after applying this process the accuracy in those cases of greater difficulty increases, going, for example, from 12% to 75% in the case of player 7.

## 4. Discussion

This article presents an innovative complete system for the diagnosis and evaluation of performance in soccer from a technical point of view. The work described is part of a more ambitious project that, based on the analysis of foot kinematics, seeks to develop support services for high-performance sports practice, the study of neurodegenerative diseases, or the development of growth, among others.

Compared to other systems used in soccer, the use of inertial sensors instead of cameras [2] is a breakthrough, reducing the cost of system implementation and avoiding the inconvenience associated with line-of-sight obstructions. Another aspect of innovation is the use of insole-embedded sensors, something that is unusual in a field where bulky, hard-wired systems with a large number of sensors distributed throughout the body are commonly seen [4,13,16,17,33]. These setups are often not comfortable for users.

The good results obtained in the detection of passes, hits, and CoDs reinforce the innovations incorporated. In practical terms, the ability to reliably detect events with such high accuracy ensures that the system can provide actionable insights for both sports performance enhancement and injury prevention. In the case of hits, the detections showed a great coincidence with the moments of impact with the ball. The algorithm used focused on making the difference in movement between the impact moments and the rest more noticeable. This precise differentiation allows clinicians and coaches to optimize striking techniques, thereby improving efficiency and reducing the risk of overuse injuries.

There is not much literature that does something similar to detect passes and shots, the most similar being the work presented in [14] and its continuations in [10,34]. These papers present a system that includes a sensor that goes in the insoles just as in our case. However, our approach differs from the one they present, since in those papers, the system shown first uses a fixed threshold to determine if the peak recorded by the sensor is relevant and then using machine learning (SVM) techniques to determine whether or not it is a pass or a shot. In our case, by using a threshold that dynamically adapts according to the player’s movement, the system eliminates the need for machine learning techniques, making it more efficient in real-world applications. Moreover, the results obtained show that our method is equally effective, being able to differentiate between events and other actions with an accuracy of 97.8% (combining the results of both tests) compared to the 96.7% of [14]. Similarly, in [10], they achieve a rate of true positives in the detection of peaks associated with hits of 95.7%, again using a fixed threshold, while our method has reached a true positives rate of 98.2%, further enhancing its applicability for monitoring and refining player performance.

It is also worth noting that the system implemented sample data at a frequency of 50 Hz, as opposed to the 100 Hz used in [14] and the 1000 Hz used in [10,34]. Obtaining similar or better results with a smaller amount of data has two upsides. On the one hand, there is no need for an additional storage unit, which is hard-wired and can be a nuisance to the player. On the other hand, since the system has been simplified, it can now be implemented on devices with limited resources.

In the case of CoD detection, there is no specific bibliography available as in previous cases. However, there are some similar studies, such as [35], which applies a comparable approach using insole inertial sensors and machine learning algorithms to basketball. Another example is [36], which detects certain gait events in the context of motor dysfunction detection. Both studies utilize machine learning-based approaches and achieve good results, but they differ from ours in that their processing is not performed in real time. Additionally, their datasets consist of only three and four subjects, which are even smaller than ours.

Regarding traditional clinical methods and similar technologies used in sports biomechanics, traditional tools, such as visual observation or motion capture systems, often require controlled laboratory settings and involve subjective interpretation or extensive post-processing times, limiting their applicability in real-world scenarios [37,38]. While pressure plates and force platforms are considered gold-standard tools for assessing plantar forces and balance, they are often stationary, expensive, and not feasible for field-based assessments [39]. Camera-based systems, frequently used in team sports, suffer from limitations such as line-of-sight occlusion and high implementation costs [40]. In contrast, the inertial sensors embedded in the insoles used in this study provide portability, objective data collection, and the ability to analyze movements in dynamic, real-world conditions. Additionally, unlike previous studies relying on fixed thresholds or machine learning for event detection, this system incorporates adaptive thresholds that account for individual variability, improving its accuracy and flexibility [41]. Table 2 summarizes the comparison presented, highlighting aspects related to user experience and the high-level information obtained.

However, the system is not without its limitations. Its exclusive reliance on inertial sensors poses challenges, such as the inability to directly measure forces, which could be mitigated in future iterations by integrating complementary technologies [41]. Furthermore, the limited number of participants used in this study may restrict the system’s generalizability. Expanding the dataset with more diverse samples represents a key avenue for future research to enhance the robustness and applicability of the system.

This demonstrates that the system presented represents a reliable tool for the detection of both passing and hitting events, as well as CoDs. In addition, being a custom system, although focused on soccer, it could easily be adapted to other areas with other requirements without the need for major changes.

## 5. Conclusions

This study presents a novel system for the diagnosis and evaluation of sports performance, specifically in soccer, based on the use of inertial sensors embedded in insoles. The system has demonstrated high accuracy in detecting events such as passes, hits, and changes of direction, with true positive rates of 98.2% for hits, surpassing previous approaches in the field. The use of adaptive thresholds and a simplified architecture that operates at 50 Hz highlights the system’s potential for practical, resource-efficient implementation without compromising precision.

From a clinical perspective, the system offers significant utility for podiatric applications. By providing detailed biomechanical insights, it facilitates the optimization of player performance and the identification of injury risk factors. This positions the system as a valuable tool not only in sports rehabilitation and prevention but also in broader podiatric contexts, such as gait analysis and the study of neurodegenerative diseases.

Future research directions should focus on expanding the dataset to include a wider variety of players, skill levels, and playing conditions to enhance generalizability. Additionally, the application of this system could be explored in other sports where similar biomechanical analyses are relevant, such as basketball, rugby, or tennis. Integrating additional sensors, such as pressure or electromyographic sensors, could provide a more comprehensive understanding of player movements and further refine injury prevention strategies. Lastly, real-time feedback capabilities could be developed to allow athletes and clinicians to make immediate adjustments during training or rehabilitation sessions.

By addressing these future directions, the proposed system can continue to evolve, ultimately contributing to both the advancement of podiatric science and the enhancement of athletic performance.

## Figures and Tables

**Table 1:** Structure of the neural networks used in the ensemble model.

Layer (Type)	Output Shape	Number of Parameters
Batch normalization	6, 35	140
Convolutional 1D	4, 32	3392
Batch normalization	4, 32	128
Convolutional 1D	2, 64	6208
Flatten	128	0
Batch normalization	128	512
Dense	24	3096
Batch normalization	24	96
Dropout	24	0
Dense	12	300
Batch normalization	12	48
Dense	2	26
Total parameters: 13,946		
Trainable parameters: 13,484		
Non-trainable parameters: 462		

**Table 2:** Summary of user experience and high-level information for sports scientists discussion.

Aspect	Other Studies	Our Approach	Discussion
User experience			
- Device Location and Size	Insoles + Storage unit on shins [14]	Insoles	Less interference with activity
- Sampling Frequency	100, 1000 Hz [10,14,34]	50 Hz	More efficient approach, longer usage time Reduced data, no need for an extra storage unit
High-Level Information for Sports Scientists			
- CoD Detection	Datasets of 3 and 4 subjects [35,36]	Dataset of 9 subjects	Better generalization due to larger dataset
- Pass/Shot Detection	Fixed threshold + ML [10,14,34,41]	Variable threshold	Simpler approach with equivalent results
- Real-Time Data	No [10,14,34]	Yes	Increased convenience for practitioners due to real-time feedback
- Applicability	Controlled laboratory settings [37,38]	Portable inertial sensors	Enables analysis of movements in real-world conditions

## Data Availability

The raw data supporting the conclusions of this article will be made available by the authors on request.

## Abbreviation

The following abbreviation is used in this manuscript:

CoD	Change of Direction
